# The Sedative Effects of Inhaled Nebulized Dexmedetomidine on Children: A Systematic Review and Meta-Analysis

**DOI:** 10.3389/fped.2022.865107

**Published:** 2022-05-20

**Authors:** Jun Lin, Chujun Wu, Dizhou Zhao, Xuhang Du, Wangzhi Zhang, Jieyu Fang

**Affiliations:** ^1^Department of Anesthesiology, The First Affiliated Hospital, Sun Yat-sen University, Guangzhou, China; ^2^Department of Anesthesiology, Guangzhou Women and Children’s Medical Center, Guangzhou Medical University, Guangzhou, China; ^3^Department of Anesthesiology, Shenzhen People’s Hospital, The Second Clinical Medical College, Jinan University, The First Affiliated Hospital, Southern University of Science and Technology, Shenzhen, China

**Keywords:** dexmedetomidine, children, sedation, surgery, spray, mucosal atomization device

## Abstract

**Background:**

Children that need surgery and medical examinations are often uncooperative, and preoperative sedation is necessary. We aimed to assess the safety and efficacy of inhaled nebulized dexmedetomidine in children for sedation that underwent medical examinations or surgery.

**Methods:**

We systematically searched PubMed, Web of science, Embase, and Cochrane library, for randomized controlled trials of Intranasal dexmedetomidine using a spray or a mucosal atomization device in children undergoing examination or elective surgery. We included all studies that analyzed the sedation efficiency of intranasal dexmedetomidine in children.

**Results:**

Ten studies with 1,233pediatric patients were included. Compared to other sedation treatments, inhaled nebulized dexmedetomidine showed similar sedation satisfaction [risk ratio RR: 1.02; 95% confidence interval (CI): 0.87–1.18; *P* = 0.83; I^2^ = 72%]. there was also no statistical difference in the success rate of separation from parents (RR: 0.96; 95% CI: 0.82–1.12; *P* = 0.58; I^2^ = 67%), and mask acceptability (RR: 1; 95% CI: 0.83–1.20; *P* = 0.99; I^2^ = 35%). But it is worth mentioning that nebulized dexmedetomidine combined with ketamine provided better sedation satisfaction (RR: 0.69; 95% CI: 0.49–0.96; I^2^ = 49%) and more satisfactory separation from parents (RR: 0.85; 95% CI: 0.74–0.97; I^2^ = 0%). Moreover, nebulized dexmedetomidine reduced the occurrences of nausea and vomiting (RR: 0.28; 95% CI: 0.15–0.51; *P* < 0.01; I^2^ = 10%) and emergence agitation (RR: 0.30; 95% CI: 0.18–0.49; *P* < 0.01; I^2^ = 0%). There are no hypotension or arrhythmia reported that required intervention in all articles.

**Conclusion:**

Compared to other premedication treatments, inhaled nebulized dexmedetomidine provided equivalent sedation satisfaction for the examination or preoperative sedation of children, but it reduced the occurrences of emergence agitation and postoperative nausea and vomiting.

## Introduction

Infants and preschool children that require surgery and imaging are often anxious, due to the separation from their parents, fear of doctors, and unfamiliar environments, and it is often difficult to obtain their cooperation (1). When a child is forcibly taken away from the parents, postoperative agitation increases, and they may experience long-term psychological trauma (2).Thus, preoperative medication is essential for children.

Preoperative medication before imaging or surgery can relieve the child’s anxiety and tension, which facilitates the child’s separation from parents, and the examination and surgery can be completed smoothly (3). To achieve this goal, various drugs act as sedative premedications in children, including midazolam, ketamine, choral hydrate, dexmedetomidine, and so on (4). Dexmedetomidine is one of the commonly used sedatives in children. It was reported that dexmedetomidine can decrease the incidence of emergence agitation and postoperative nausea and vomiting events in children undergoing surgery under general anesthesia (5). Due to small blood vessels and physical agitation, the intravenous route most commonly used by clinicians is technically challenging in children (6). Furthermore, oral formulations have a slow onset of action and frequently cause adverse gastrointestinal reactions. A potential alternative is an intranasal approach premedication, which avoids puncture pain and causes few adverse gastrointestinal reactions (7).

Inhalation of a nasal aerosol is the latest route of premedication for children. The benefits of aerosolized release include less drug loss in the oropharynx, higher cerebrospinal fluid levels, better patient acceptance, and better sedation (8). Dexmedetomidine is a highly selective alpha-2 adrenal receptor agonist, often given as a premedication. It acts as a sedative, with analgesic and anxiolytic characteristics. It inhibits sympathetic nerve activity, promotes hemodynamic stability, and causes only slight respiratory depression (9). Dexmedetomidine showed anti-inflammatory properties as well. However, as a new preoperative medicine, there are no literature reviews on the relative effectiveness of nebulized inhaled dexmedetomidine compared to other intranasal drips or oral premedications.

The present study aimed to determine the sedative effectiveness of inhaled nebulized dexmedetomidine as a pediatric premedication. This systematic review and meta-analysis included randomized controlled trials (RCTs) that tested the efficacy of inhaled intranasal dexmedetomidine aerosol, administered with either a nasal mucosal atomizer device or a professional medical nebulizer.

## Materials and Methods

We followed the Cochrane methodology for systematic reviews. Our results are reported according to the Preferred Reporting Items for Systematic Reviews and Meta-Analyses (PRISMA) statement (10). The study was registered in the PROSPERO database (number: CRD42021271470).

### Search Strategy

Two independent reviewers (Jun Lin and Jieyu Fang) systematically searched relevant literature from databases including PubMed, Embase, Web of science, and Cochrane Library, from the date of inception until October 2021. The keywords used for searching were: dexmedetomidine, infant, children, pediatric, intranasal, nebulized, atomizer; we searched text words and Medical Subject Headings (Mesh) terms (see Appendix). In addition, we conducted a tangential electronic exploration of related articles (i.e., with links to related articles in reference lists). There were no language restrictions but researches were limited to human studies.

### Eligibility and Inclusion Criteria

Randomized controlled trials (RCTs) were included in the meta-analysis. The criteria for inclusion in these trials and studies were as follows: (1) pediatric patients (<12 years old) that had undergone elective operations and diagnostic procedures; (2) interventions with intranasal dexmedetomidine as a premedication, administered with a nasal mucosal atomizer device, nebulizer, or spray (we did not limit the range of the specific administered doses); and (3) comparisons with children who received other sedative drugs. In addition, the included studies had to include at least one of the following primary outcomes: (1) the number of children with satisfactory sedation, defined as acceptable venipuncture, acceptable diagnostic procedures, and acceptable operations; (2) the number of children with satisfactory separation from parents; and (3) the number of children with satisfactory mask acceptance. Or included studies had at least one of the following secondary outcomes: (1) the number of children with emergence agitation, (2) onset of sedation, (3) recovery time, and (4) the number of children with adverse events, including bradycardia, hypoxia, hypotension, nausea, and vomiting.

### Exclusion Criteria

Studies that met any of the following conditions were excluded: (1) systematic reviews, (2) case reports, or (3) missing the specific number of satisfactory sedations.

### Study Selection and Data Extraction

Two independent reviewers used standard forms to conduct the literature search and data extraction. Each reviewer’s results were cross-checked by the other reviewer. After selecting articles that met the inclusion and exclusion criteria, the reviewers eliminated all duplicate articles. Next, the two reviewers initially screened the articles by the title and abstract, and then included all eligible studies based on the full text.

Data were extracted to a Microsoft Excel spreadsheet to organize the following information (Table 1): first author, publication year, country, range of age, American Society of Anesthesiologists status, type of surgery/procedure, intervention route, drug dosage, sample size, and outcome. In accordance with the recommendations of the Cochrane Handbook for Systematic Reviews of Interventions, we conducted a separate, pairwise comparison for a multi-arm study, and the shared control group was nearly evenly distributed in the comparison.

**TABLE 1:** The characteristics of the included studies.

Study	Race	Range of age	ASA satus	Type of surgery/procedure	Intervention route	Dosage	Sample size	Outcome
Sado-Filho et al. (12)	Brazil	1–7 year	I–II	Dental procedures	DEX by MAD DEX + Ketamine by MAD	2.5 μg/kg 2 μg/kg + 1 mg/kg	D:44 D + K:44	➀➄➅➆
Azizkhani et al. (36)	Iran	1–6 year	/	Computerized tomography (CT)	Dex by MAD Midazolam by MAD	3 μg/kg 0.3mg/kg	D:78 M:68	➄➅
Sathyamoorthy et al. (13)	United States	>5 year	I-II	Dental procedures	DEX by MAD oral midazolam	2 μg/kg 0.5 mg/kg	D:36 M:37	➀➂➆
Abdel-Ghaffar et al. (14)	Egypt	3–7 year	I-II	Bone marrow biopsy	nebulised DEX nebulised ketamine nebulised midazolam	2 μg/kg 2 mg/kg 0.2 mg/kg	D:30 M:30 K:30	➀➁➂➃➅➆
Miller et al. (19)	United States	3–24 month	I-II	Transthoracic echocardiographic	DEX by MAD oral pentobarbital	2.5 μg/kg 5mg/kg	D:140 P:139	➀➃➄➆
Yuen et al. (18)	China	<4 year	/	Computerized tomographic(CT)	DEX by MAD oraL choral hydrate	3 μg/kg 50mg/kg	D:87 C:107	➀➆
Qiao et al. (16)	China	2–6 year	I-II	Eye surgery	nebulized DEX oral ketamine nebulized DEX + oral K	2.5 μg/kg 6mg/kg 2μg/kg + 3mg/kg	D:42 K:41 D + K:41	➀➄➅➆
Cao et al. (11)	China	3 month–3 year	I-II	Ophthalmic examination	DEX by MAD oral chloral hydrate	2 μg/kg 80mg/kg	D:71 C:70	➀➁➄➅➆
Neville et al. (15)	United States	1–5 year	/	Laceration repair	DEX by MAD midazolam by MAD	2 μg/kg 0.4 mg/kg	D:20 M:18	➀➆
Zanaty et al. (17)	Egypt	3–6 year	I-II	Dental surgery	nebulized DEX nebulized ketamine nebulized D and K	2 μg/kg 2 mg/kg 1 μg/kg + 1mg/kg	D:20 K:20 DK:20	➀➁➂➃➅➆

➀ *satisfactory sedative,* ➁ *satisfactory separation from parents,* ➂ *satisfactory mask acceptance,* ➃ *emergency agitation,* ➄ *onset of sedation,* ➅ *recovery time,* ➆ *adverse events. DEX, dexmedetomidine, MAD, mucosal atomization device; D, dexmedetomidine; K, ketamine; M, midazolam; P, pentobarbital; C, chloral hydrate.*

### Risk-of-Bias Assessment

We used the Cochrane risk of bias tool to assess biases in RCTs. RCTs were appraised in seven domains: random sequence generation, allocation concealment, blinding, outcome assessment, incomplete outcome data, selective reporting, and other biases. Each potential source of bias was graded as low, unclear, or high. When the two reviewers’ assessments were inconsistent, they reached agreement through discussion with each other or by consulting with a third investigator.

Risk-of-bias assessment was conducted with Review Manager 5.4, provided by the Cochrane Collaboration.

### Statistical Analysis

Statistical analyses were performed using R [R version 4.1.1]. Continuous outcome data are presented as the standardized mean difference (SMD) between studies, and the corresponding 95% confidence interval (CI). For studies reporting continuous outcome data reported as the median and interquartile range, we estimated the overall mean and standard deviation (SD), based on those values. The risk ratio (RR) and its corresponding 95% CI were calculated to analyze dichotomous data. In each analysis, we assessed heterogeneity among studies with the I-squared (I^2^) test, where values greater than 50% indicated substantial heterogeneity. If significant heterogeneity (I^2^ > 50) emerged, the subgroups analysis was performed by with subgroups defined by the different drugs administered (i.e., midazolam, ketamine, and chloral hydrate), method of control group intervention (i.e., oral, drops, or nebulized). Moreover, sensitivity analysis was performed to explore heterogeneity for the primary outcome by omitting each study individually and the random effects model was applied. Finally, funnel plot was used to detect publication bias.

*P*-values < 0.05 were considered statistically significant.

## Results

### Literature Search

**FIGURE 1:**
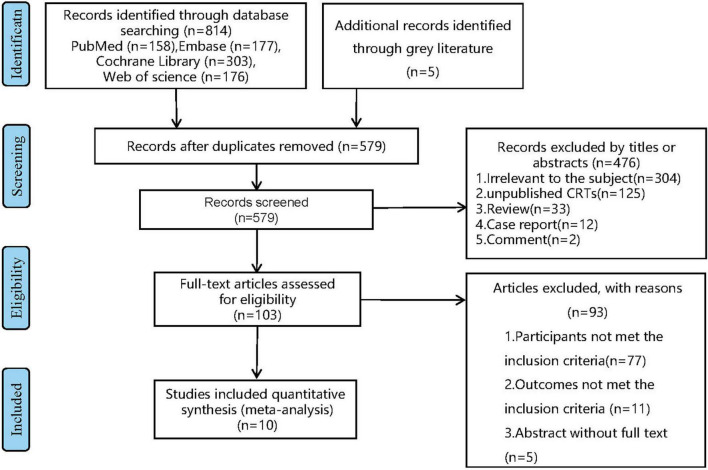
PRISMA flow chart for selection study. CRTs, clinical research trials.

Through the above search strategy, we identified a total of 814 articles from database PubMed, Embase, Web of science, and Cochrane Library. After removing duplicate articles, we screened article titles and abstracts and excluded 304 articles with irrelevant subjects, 125 unpublished clinical trials, 33 reviews, 12 case reports, and 2 comments by screening their titles and abstracts. A total of 93articles were excluded by full-text reviewing, including 77 that did not meet the inclusion criteria, 11 that reported uncorrelated outcomes, the rest of articles were unable to obtain full text. Finally, 10 eligible articles. (The articles selection process is displayed in Figure 1).

### Study Characteristics

The 10 RCTs analyzed included a total of 1,233 children, ranging in age from 3 months to 7 years. Among them, there are four studies compared nebulized dexmedetomidine to midazolam, three studies compared nebulized dexmedetomidine to ketamine, two studies compared nebulized dexmedetomidine to chloral hydrate, and one study compared nebulized dexmedetomidine to pentobarbital. Three studies also compared nebulized dexmedetomidine in combination with ketamine to nebulized dexmedetomidine alone. In addition, six of the ten studies involved children undergoing diagnostic procedures, while the other four studies involved children undergoing elective surgery. The primary outcomes were “number of children with satisfactory sedation” (9 studies), “number of children with satisfactory separation from parents” (3 studies) and “number of children with satisfactory mask acceptance” (3studies), The characteristics of the studies that were included in our meta-analysis are summarized in Table 1.

### Risk of Bias Assessment

**FIGURE 2:**
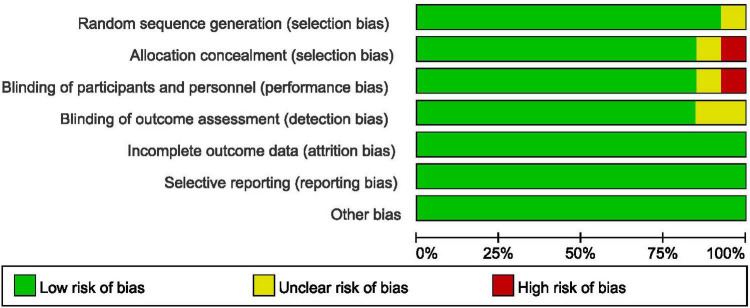
Risk of bias graph.

**FIGURE 3:**
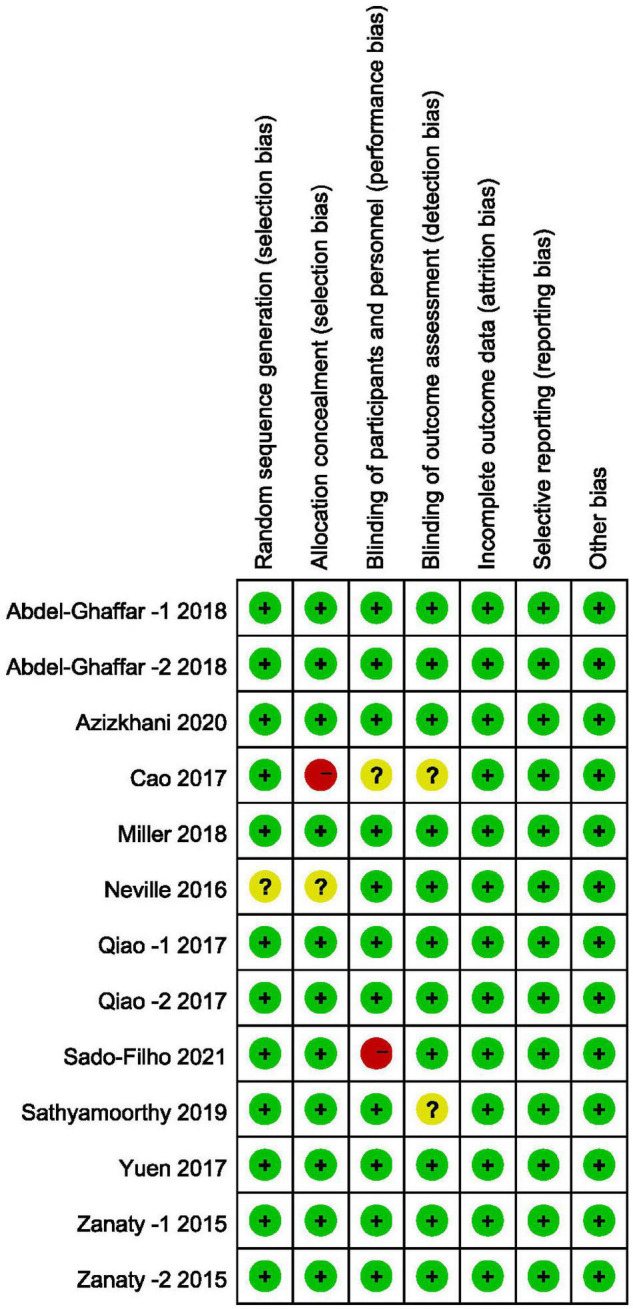
Risk of bias summary.

In accordance with the risk of bias assessment method mentioned in the appeal, 90% (9/10) of studies performed an adequate method of random sequence generation, and 8 studies reported detailed allocation methods, including opaque envelopes and pharmacy-controlled random medication distributions. However, in the study by Cao et al. (11) the use of odd and even as an allocation method was assessed as high risk. Eight studies used the methods of blinding to patients and researchers. In the study by Sado-Filho et al. (12), the anesthesiologists and surgeons were not blinded to the medication. Eight studies used the methods of blinding to outcome assessment. Moreover, all studies were considered to have no other biases. Figures 2, 3 illustrate in detail the risk of bias assessment results.

### Outcomes of Pooled Studies

#### Satisfactory Sedation

**FIGURE 4:**
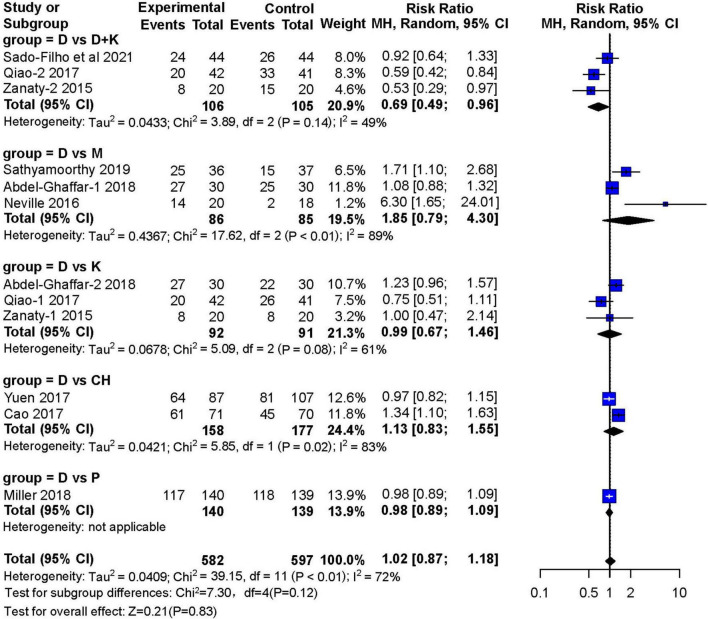
Meta-analysis of nebulized dexmedetomidine for sedation satisfaction.CI, confidence interval; df, degress of freedom; M-H, Mantel-Haenszel.

Nine studies reported the number of children who achieved satisfactory sedation. Three RCTs (13–15) with 171 children showed that sedation with nebulized dexmedetomidine was not significantly different (RR: 1.85; 95% CI: 0.79–4.30; I^2^ = 89%) from sedation with midazolam (two studies used inhaled midazolam and one used oral midazolam) (Figure 4). Three RCTs (14, 16, 17) with 183 children compared nebulized dexmedetomidine to ketamine (two administered with nebulization). They showed no significant difference in sedation(RR:0.99; 95% CI: 0.67–1.46; I^2^ = 61%). Furthermore, no significant differences in sedation were found in two studies (11, 18) that compared nebulized dexmedetomidine and oral chloral hydrate (RR: 1.13; 95% CI: 0.83–1.55; I^2^ = 83%), or in one RCT (19) that compared nebulized dexmedetomidine and oral pentobarbital (RR: 0.98; 95% CI: 0.89–1.09). However, three RCTs (12, 16, 17) with 211 children showed that satisfactory sedation of nebulized dexmedetomidine and ketamine was better (RR: 0.69; 95% CI: 0.49–0.96; I^2^ = 49%).

**FIGURE 5:**
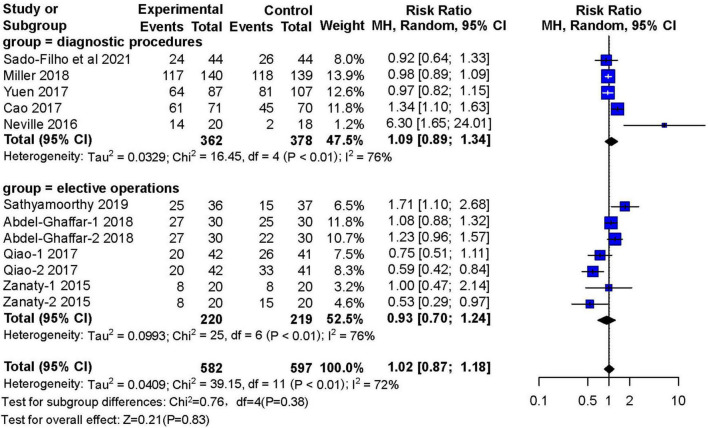
Meta-analysis of nebulized dexmedetomidine for sedation satisfaction in diagnostic procedures and elective operations.CI, confidence interval; df, degress of freedom; M-H, Mantel-Haenszel.

Five studies (*n* = 740) compared the sedative effects of nebulized dexmedetomidine with other drugs in diagnostic procedures. There was no significant difference in sedation satisfaction (RR: 1.09; 95% CI: 0.89–1.34; I^2^ = 76%). Four studies (*n* = 439) reported the number of people who received nebulized dexmedetomidine premedication for sedation before elective surgery. Compared with other preoperative sedative drugs, nebulized dexmedetomidine can achieve the same sedative effects (RR: 0.93; 95% CI: 0.70–1.24; I^2^ = 76%) (Figure 5).

#### Satisfactory Separation From Parents

**FIGURE 6:**
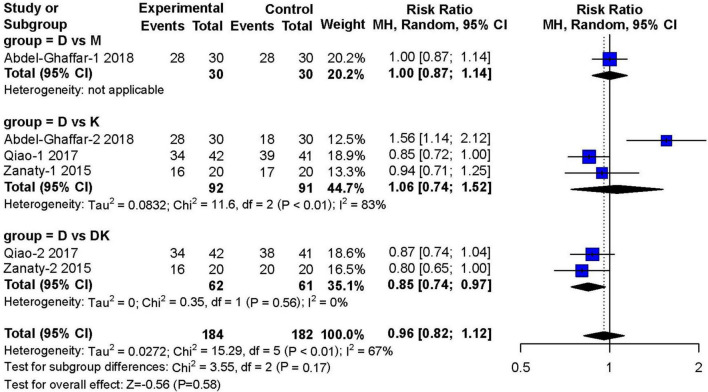
Meta-analysis of nebulized dexmedetomidine for satisfactory separation from parents.CI, confidence interval; df, degress of freedom; M-H, Mantel-Haenszel.

Three studies (14, 16, 17) (*n* = 466) investigated satisfactory separation from parents after 30 min of premedication. Compared to other types of sedatives, dexmedetomidine showed no obvious advantage in satisfactory separation from parents (RR: 0.96; 95% CI: 0.82–1.12; *P* = 0.58). However, there was moderate heterogeneity across studies (I^2^ = 67%) (Figure 6).

In the subgroup analysis, dexmedetomidine combined with ketamine was superior to dexmedetomidine alone in achieving satisfactory separations of children from their parents (RR: 0.85; 95% CI: 0.74–0.97; I^2^ = 0%). However, the study by Qiao et al. (16) tested nebulized dexmedetomidine combined with oral ketamine, instead of applying both drugs in a nebulized form. Analysis of the three literatures found that nebulized DEX combined with ketamine can achieve better sedative effect than nebulized DEX alone (70 vs. 49.1%), of which two used the dose of DEX is 2 μg/kg, one article is 1 μg/kg; the dose of nebulized ketamine used in two articles is 1 mg/kg, and the dose of oral ketamine was 3 mg/kg in one article.

#### Satisfactory Mask Acceptance

**FIGURE 7:**
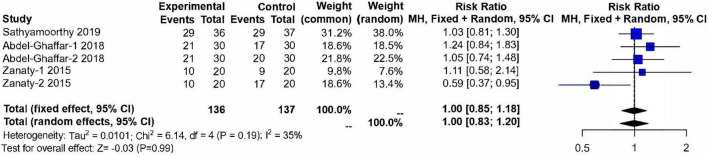
Meta-analysis of nebulized dexmedetomidine for satisfactory mask acceptance. CI, confidence interval; df, degress of freedom; M-H, Mantel-Haenszel.

Three studies (13, 14, 17) (*n* = 273) evaluated the acceptability of anesthesia masks after 30 min medication. These studies included 137 children in the control group and 136 children in the nebulized dexmedetomidine group. No significant difference was found between groups (RR:1.00; 95% CI: 0.83–1.20; *P* = 0.99; I^2^ = 35%) (Figure 7).

#### Emergence Agitation

**FIGURE 8:**
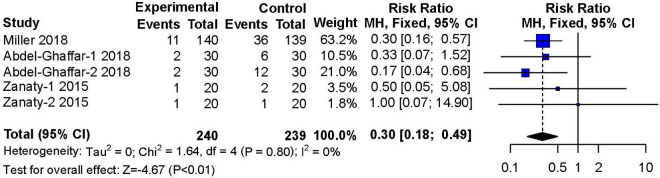
Meta-analysis of nebulized dexmedetomidine for emergence agitation. CI, confidence interval; df, degress of freedom; M-H, Mantel-Haenszel.

Three studies (14, 17, 19) reported on the occurrence of agitation during the recovery period after surgery or procedures. These studies included 240 patients in the nebulized dexmedetomidine group and 239 patients in the control group. The incidence of emergence agitation was significantly lower in the nebulized dexmedetomidine group than in the control group (RR: 0.30; 95% CI: 0.18–0.49; *P* < 0.01), and almost no heterogeneity(I^2^ = 0%) (Figure 8).

#### Onset of Sedation

**FIGURE 9:**
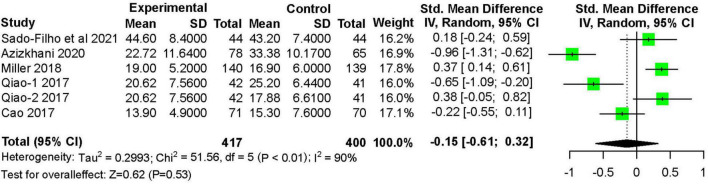
Meta-analysis of nebulized dexmedetomidine for onset of sedation. CI, confidence interval; df, degress of freedom; M-H, Mantel-Haenszel.

Five studies (11, 12, 16, 19–22) recorded the onset time of sedation after treatment. These studies included 417 children in the nebulized dexmedetomidine group and 400 children in the control group. Nebulized dexmedetomidine has no statistical difference in onset time compared with other treatments (SMD: −0.15 95% CI: −0.61 to 0.32; *P* = 0.53; I^2^ = 90%) (Figure 9).

#### Recovery Time

**FIGURE 10:**
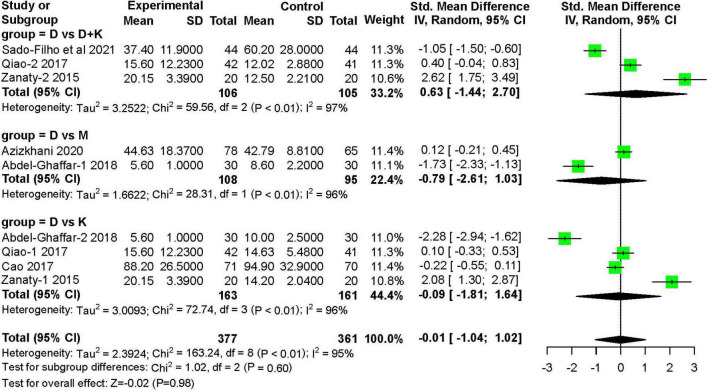
Meta-analysis of nebulized dexmedetomidine for recovery time. CI, confidence interval; df, degress of freedom; M-H, Mantel-Haenszel.

Six studies (11, 12, 14, 16, 17) recorded the recovery time after treatment. These studies included 377 children in the nebulized dexmedetomidine group and 361 children in the control group. The recovery time was not significantly different between groups (SMD: −0.01; 95% CI: −1.04 to 1.02; *P* = 0.98; I^2^ = 95%) (Figure 10).

#### Nausea and Vomiting

**FIGURE 11:**
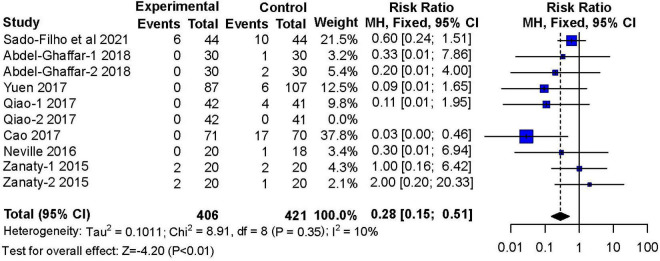
Meta-analysis of nebulized dexmedetomidine for postoperative nausea and vomiting. CI, confidence interval; df, degress of freedom; M-H, Mantel-Haenszel.

Seven studies (11, 12, 14–18) reported adverse effects of nausea and vomiting These studies included 406 children in the nebulized dexmedetomidine group and 421 children in the control group. The incidence of nausea and vomiting during the recovery period was lower with nebulized dexmedetomidine sedation compared to other premedication treatments (RR: 0.28; 95% CI: 0.15–0.51; *P* < 0.01; I^2^ = 10%) (Figure 11).

## Discussion

Pediatric sedation has always been a challenge for anesthesiologists and pediatricians, particularly when venous access is difficult. Moreover, venipuncture is invasive, despite the rapid onset of intravenous drug effects. Therefore, many other routes of administration are preferential for children, such as oral, sublingual, buccal, nasal drops, and nasal atomization. In recent years, literature reviews have shown that intranasal dexmedetomidine premedication was safe and effective in children, with few side effects (23). Dexmedetomidine is administered through the nasal mucosa, which is an easy, non-invasive route with high bioavailability (83.8%) (24). There are two ways to administer drugs in the nose, one is with a syringe drip, and the other is with an atomized drug sprayed into the nasal mucosa. A previous study on the pharmacodynamics and pharmacokinetics of dexmedetomidine showed that atomization and nasal drops had similar bioavailability and achieved a similar degree of sedation (25). However, the effectiveness and safety of atomized dexmedetomidine administration into the nasal mucosa in children remain uncertain.

This meta-analysis revealed that administering intranasal atomized dexmedetomidine into the nasal mucosa as a premedication for pediatric patients did not show any obvious advantage in the achievement of satisfactory sedation or separation from parents, compared to other premedication regimes, like midazolam (26). Similarly, other previous studies on intranasal dexmedetomidine showed that mask acceptance was not significantly different with nebulized dexmedetomidine compared to other intranasal or oral preoperative medicines. Additionally, nebulized dexmedetomidine was as effective as other drugs for sedation, both before diagnostic procedures and before elective surgery in children. However, our analysis found that, when nebulized dexmedetomidine was combined with oral or nebulized ketamine, it achieved better sedation and more frequently facilitated the separation from parents. This is consistent with the conclusion of Qian’s research (27). Dexmedetomidine produces sedative, analgesic, and anti-sympathetic effects by acting on α2 receptors on locus coeruleus (28). Its sedative effect is similar to the non-eye-moving, rapid phase of physiological sleep; the child is easy to wake; and it does not induce obvious respiratory depression (29). Ketamine has the characteristics of anesthesia, sedation and analgesia by antagonizing the N-methyl-D-aspartate receptor. Consequently, it has become one of the most commonly used drugs in clinical pediatric anesthesia (30). Because dexmedetomidine and ketamine act through different mechanisms, this combination may enhance the sedative effect, but avoid the adverse reactions of hypotension and bradycardia. However, only 3 studies were available on the combination of these two drugs. Thus, the sample size was too small to confirm this conclusion. More studies are needed to investigate this hypothesis.

Compared to other premedication regimes, nebulized dexmedetomidine was associated with significantly lower incidences of emergence agitation and postoperative nausea and vomiting. Due to the lack of cognitive development, fear of the environment, and sensitivity to pain, children have a high incidence of restless during the recovery period (31). When preschool children were given general anesthesia with sevoflurane, the incidence of emergence agitation varied from 10 to 66% (32). The reduction in emergence agitation with nebulized dexmedetomidine was attributed to its ability to induce a natural sleep state; and in addition, it had an analgesic effect and did not cause respiratory tract irritations (33).

In addition, we searched three articles that tested different doses of nebulized dexmedetomidine. It is worth mentioning that Jin et al. (34) applied nebulized dexmedetomidine before plastic surgery to 41 children, aged 3–6 years, and found that the 2 μg/kg dose was more effective than the 1 μg/kg dose in achieving satisfactory separations from parents. In another study, Anupriya et al. (22) administered aerosolized dexmedetomidine preoperatively to 59 children, aged 1–8 years, before circumcision or herniotomy. They analyzed different age groups of children and found that 2–3 μg/kg inhaled nebulized dexmedetomidine was safe and effective for sedation and that 3 μg/kg increased the frequency of satisfactory parental separation among younger children. In Dhingra’s study, nebulized dexmedetomidine at a dose of 3.5 μg/kg has a higher sedation success rate in postoperative follow-up examinations in children with glaucoma (37). However, according to the current research, the most appropriate doses for children remain uncertain. Hence, further studies are needed to test different doses of nebulized dexmedetomidine in children of different ages.

In terms of onset time, nebulized dexmedetomidine did not provide a faster onset time and recovery time, compared to other drugs. As for other side effects, although nebulized dexmedetomidine could cause mild drops in blood pressure and heart rate, no patients required additional drug intervention. No study reported a dangerous drop in oxygen saturation.

The inhalation route may be a better way of sedation, because it is easy to implement, it does not require intravenous cannulation or injection, and it has relatively high bioavailability (35). In addition, nebulization technology is convenient for medical staff and more acceptable to patients. Thus, nebulized dexmedetomidine may be the first choice for pediatric sedation.

Based on I^2^ estimates, no significant heterogeneity was detected for mask acceptance or for the incidences of emergence agitation or nausea and vomiting. However, significant heterogeneity was observed for sedation satisfaction, satisfaction in the separation from parents, and the onset and recovery times. This heterogeneity might be explained by the diversity of medications used in our patient population. Consequently, we constructed a random effects model and performed subgroup analyses; however, a moderate degree of heterogeneity remained. This result might have been due to different sedation scoring standards in different studies and different doses of a given drug between studies. The study quality analysis showed that some studies were not implemented strictly in accordance with an RCT, which increased the selection bias. In addition, several studies included children that required general anesthesia for surgery, and some included children that were only examined. After general anesthesia, the recovery time of children after general anesthesia will be prolonged; consequently, these differences with the extension of the anesthesia time, might explain the large heterogeneity in recovery times.

This meta-analysis had several limitations. First, the number of included studies was not sufficient, particularly for some parameters, such as mask acceptance, which only comes from three articles with a total sample size of 136/137 patients. This limitation could have reduced the reliability of the results and increased the publication bias. Second, of the 10 trials included in our analysis, threetrials originated from China and three studies originated from America. Thus, we may have introduced another source of publication bias. Third, although we conducted subgroup analyses, we did not identify the exact cause of the heterogeneity. Potential causes included differences in the types of procedures, administration routes, premedication doses, and age groups; therefore, future trials are warranted that focus on specific types of surgery-related sedation with nebulized dexmedetomidine. Lastly, safe doses and long-term potential side effects remain unknown for inhaled aerosol dexmedetomidine in children. More studies are needed to evaluate safety.

## Conclusion

In summary, current evidence has suggested that nebulized dexmedetomidine can provide satisfactory sedation and reduce emergence agitation and postoperative nausea and vomiting. Moreover, nebulized dexmedetomidine combined with ketamine could achieve better sedation than dexmedetomidine alone. However, further research is required to determine the optimal doses and the optimal drug combination for inhaled nebulized dexmedetomidine.

## Data Availability Statement

The original contributions presented in the study are included in the article/supplementary material, further inquiries can be directed to the corresponding author.

## Author Contributions

JL and CW conceived and designed the study. JL, CW, and DZ wrote the manuscript. JF reviewed and edited the manuscript. All authors listed have made a substantial contribution to the acquisition, analysis, and interpretation of the data for the work and read and approved the final manuscript.

## Conflict of Interest

The authors declare that the research was conducted in the absence of any commercial or financial relationships that could be construed as a potential conflict of interest.

## Publisher’s Note

All claims expressed in this article are solely those of the authors and do not necessarily represent those of their affiliated organizations, or those of the publisher, the editors and the reviewers. Any product that may be evaluated in this article, or claim that may be made by its manufacturer, is not guaranteed or endorsed by the publisher.
